# Concentrations of Aerosol Numbers and Airborne Bacteria, and Temperature and Relative Humidity, and Their Interrelationships in a Tie-Stall Dairy Barn

**DOI:** 10.3390/ani9121023

**Published:** 2019-11-24

**Authors:** Md. Aminul Islam, Atsuo Ikeguchi, Takanori Naide

**Affiliations:** 1 Department of Agricultural and Environmental Engineering, United Graduate School of Agricultural Science, Tokyo University of Agriculture and Technology, 3-5-8 Saiwai-cho, Fuchu-shi, Tokyo 183-8509, Japan; 2 Department of Medicine, Faculty of Veterinary Medicine & Animal Science, Bangabandhu Sheikh Mujibur Rahman Agricultural University, Gazipur 1706, Bangladesh; 3 Department of Environmental Engineering, Faculty of Agriculture, Utsunomiya University, 350 Minemachi, Utsunomiya 321-8505, Japan; ike14000@gmail.com; 4 Earth Environmental Service Co., Ltd., 17 Kanda-konyacho, Chiyodaku, Tokyo 101-0035, Japan; taka0529n@gmail.com

**Keywords:** aerosol numbers, airborne bacteria, temperature, relative humidity, tie-stall barn, dairy cows, summer season

## Abstract

**Simple Summary:**

Aerosol particles are important elements of atmospheric pollution. Livestock barns are some of the most crucial anthropogenic sources of generation and emission of aerosol particles to the surrounding environment. Along with environmental pollution, these particles can cause numerous diseases in humans and animals. Besides causing diseases, these particles are said to have a significant role in the transmission of infectious diseases. Microorganisms that bind to aerosol particles are known as bioaerosols, which ultimately spread diseases in and between farms. Environmental factors such as temperatures (temp.) and relative humidity (RH) may have significant effects on aerosol particles and airborne bacteria. The present study was undertaken to find out which factors have more significant effects on the regulation of aerosol particles and airborne bacteria in the dairy environment. In the present study, we found that temperatures have profound effects on the regulation of aerosol numbers and various airborne bacteria. This temperature-dependent emission inventory of aerosol particles and various airborne bacteria will play a crucial factor in the mitigation of aerosol particles and various airborne bacteria in the dairy environment.

**Abstract:**

Aerosol particles and airborne microorganisms are crucial factors of indoor air quality. The purpose of the present study was to evaluate the interrelationships among aerosol numbers, various types of airborne bacteria, temperature, and relative humidity (RH) to decide which parameters have more significant relationships among them. The concentrations of aerosol numbers, airborne total aerobic bacteria, *Staphylococcus aureus* (*S. aureus*), and *Escherichia coli* (*E. coli*) in the indoor air, as well as indoor and outdoor temperatures and RH, were assessed each week for a total of 20 days in a tie-stall dairy barn during the summer season in Tochigi, Japan. The mean concentrations of the fine aerosol numbers (0.3–2.0 µm) were greater than the mean concentrations of coarse aerosol numbers (5.0–10.0 µm). Among the airborne total aerobic bacteria, the mean concentration of airborne *S. aureus* was higher compared with airborne *E. coli*. More significant positive associations were found between outdoor environmental temperatures and aerosol numbers rather than indoor temperatures and aerosol numbers. All three types of airborne bacteria were associated with both outdoor and indoor environmental temperatures. These findings are crucial in the mitigation of aerosol numbers and airborne bacteria in the indoor air of dairy barns.

## 1. Introduction

Along with nutrition supplements, improving the environment around the animals is a prerequisite for improving productivity in dairy cows. Improved housing is an essential factor of advanced environmental management in dairy cows. Excellent air quality is the crucial criterion for improved housing and a comfortable environment for animals and humans [1,2,3].

Aerosol particles and airborne microorganisms are considered essential components of air quality. Aerosols are fine solid particles or liquid droplets that are suspended in the air or gas [4]. Aerosol particles can carry abiotic inorganic substances such as metals (Zn, As, V) and organic substances (polycyclic aromatic hydrocarbons, PAHs) that are carcinogenic and promote the progression of cancer [5]. At present, aerosol particles are also considered as an essential contributor to climate change, the most severe environmental challenge humanity has to face, which threatens the well-being of the next generation.

Microorganisms can also be associated with aerosol particles because of their smaller size. The size of the viruses varies from 0.02 to 0.3 μm, and the size of bacteria varies from 0.5 to 10 μm in their naked form. When the aerosol particles consist of biotic compounds (viruses, bacteria, fungi, and pollen), then these aerosol particles are known as bioaerosols. Besides these, fine aerosol particles could also induce infection of microorganisms [6]. A recent study also found strong links among microorganisms infection, inflammation, and apoptosis in cell response to PM_2.5_ (particulate matter 2.5 µm or less in diameter) carried microbes [7].

*Mycobacterium tuberculosis*, the causative agent of tuberculosis, has been reported in small droplet nuclei, and patients have generated bacteria-laden aerosols in a diameter range of 0.65–4.7 μm during coughing [8]. An aerosol-laden jet, led by a characteristic vortex, can penetrate an impressive distance into the surrounding ambient air before finally mixing outwards [9]. The expired droplets can travel 1.5–2.0 m [10], and the presence of turbulence greatly enhances the droplet spread [11]. Pathogenic aerobic bacteria, including *Staphylococcus aureus* and *Escherichia coli*, are crucial for the maintenance of healthy dairy cows. All three types of bacteria cause numerous infectious diseases in dairy cows. Besides causing diseases, *E. coli* is a significant potential endotoxin carrier. The barn air microorganisms can also contaminate freshly milked milk by post-secretory contamination [12], which ultimately will cause severe consequences from the perspective of public health.

With the diversity of livestock production systems, the aerosol particles from livestock barns can originate from a wide variety of sources, and this results in aerosol particles from livestock barns being very heterogeneous in composition and morphology [4]. The generation of the aerosol particles, its suspension in the air, and its release to the outside of livestock barns depend on the kind of housing and feeding, animal type, and environmental factors related to climatic conditions [13]. Despite their heterogeneity, the aerosol particles from livestock barns are unique not only because of its high organic content (above 90%), and they comprise many sizes, shapes, densities, and chemical substances. They can also act as a carrier of other substances such as odorous compounds and gases [14], which are generated and available inside livestock barns. Exposure to the aerosol particles from the livestock barns and its compounds can cause death in farm animals [15], pose severe damage to the respiratory health of farmers and people living in the vicinity of the farms, and cause adverse environmental effects [16,17,18]. However, the detailed mechanisms of the aerosol particle-mediated human and animal diseases are still unclarified [19].

Physical characteristics of the environment, such as the temperatures and RH, are essential [20] for regulating the aerosol particles and airborne microorganisms. The airborne microorganisms are exposed to meteorological factors, particularly to temperatures, RH, and solar radiation [21]. These factors may have significant effects on the survival of these microorganisms. Furthermore, airborne microorganisms might be protected from outside influences by aerosol particles coagulated with viable particles. Besides, temperatures and RH also have effects on the aerosol particles [22,23]. The microbial activity in the air will be inhibited if the RH is too low because a dry environment will depress the metabolism and physiological activities of the microorganisms [24]. It was reported that most of the Gram-negative bacteria in the air survived longer in lower RH conditions. In contrast, Gram-positive bacteria were more likely to survive under higher RH conditions [25]. Except differences between species of microorganisms, meteorological conditions presumably will determine to a large extent for the survival time of microbial pathogens and the distance they can bridge to infect other farms and humans.

Airborne transmission has been responsible for the epidemics of highly infectious diseases in intensive livestock production systems. In such transmission, the pathogenic microorganisms may associate with aerosol particles. However, how airborne transmission spreads infectious diseases between the farms, and the relationships among microorganisms, aerosol particles, and environmental factors remain unclear [19]. Furthermore, most of the studies in the dairy barns were aimed at measuring the prevalence or incidence of various diseases of dairy cows. Very few studies have been undertaken for measuring either various types of airborne bacteria [2,26,27] or aerosol particles [13,15,28] in dairy barns. None have been undertaken to investigate the concentrations of the aerosol particle numbers and various types of pathogenic airborne bacteria, and their dependence on the temperatures and RH, in tie-stall dairy barns. The first aim of the present study was to elucidate the aerosol number concentrations, concentrations of the important airborne pathogenic bacteria, and temperatures and RH in the indoor air of dairy barns. The second aim was to perform statistical analyses to look for relationships among the aerosol particle numbers, different types of airborne bacteria, and temperatures and RH to determine which factors were the most significant for the regulation of different sizes of the aerosol particle numbers and various types of airborne bacteria in the indoor air in a tie-stall dairy barn during the summer season.

## 2. Materials and Methods

### 2.1. Dairy Barn

**Figure 1:**
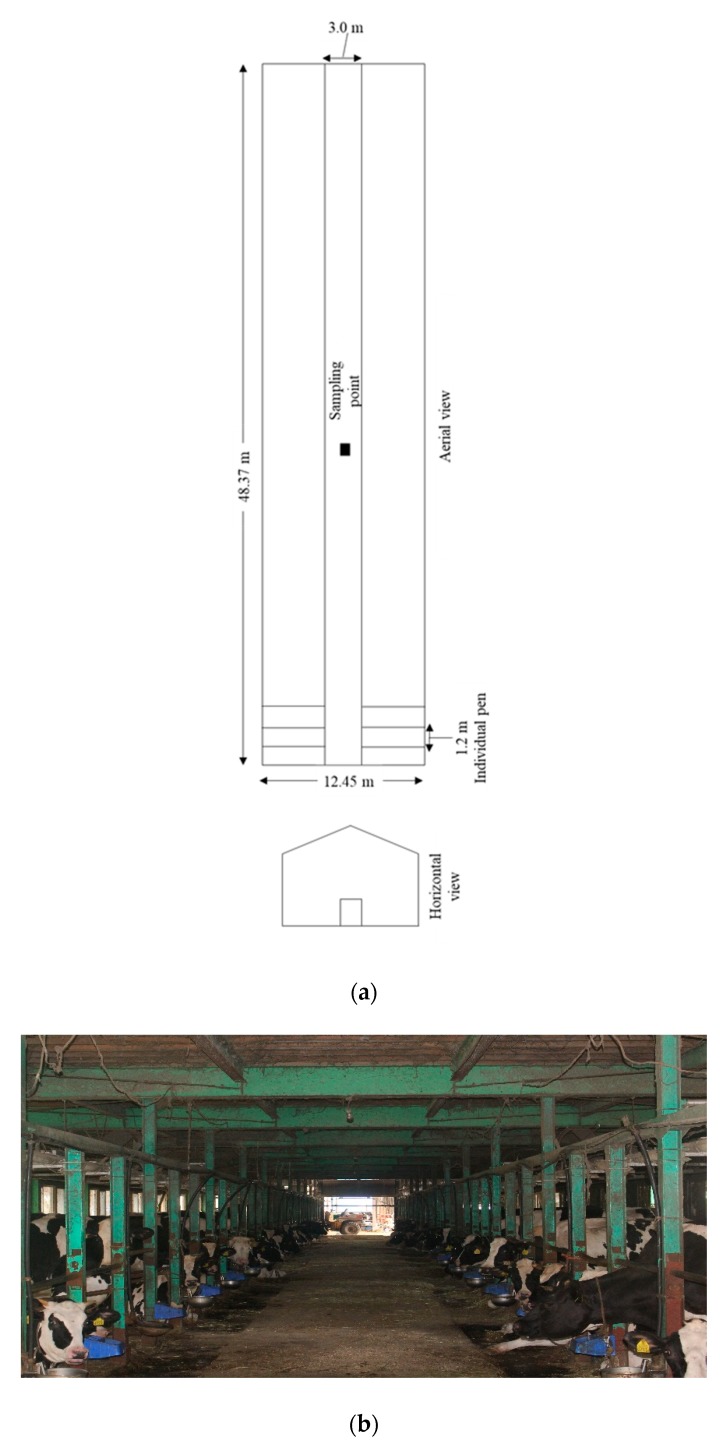
(**a**) Schematic diagram of the tie-stall dairy barn. (**b**) The tie-stall dairy barn.

The research was conducted in a tie-stall dairy barn in Shirahisa, Nakagawamachi, Nasugun, Tochigi, Japan. The temperatures and RH were influenced by the weather outside. The ventilation of the barn was natural, with an additional 14 fans divided into two rows in the house. The area of the barn was 602.21 m^2^. There was a central alley of 3.00 m^2^. There were 76 pens in the barn that were divided into two rows. The width of each pen was 1.2 m. The floor of the barn consisted of a rubber mat and without any litter. The general features of the barn are shown in Figure 1a,b. The topography of the Nasugun is greatly influenced by the numerous hills of the region. There were about 65 dairy cows in the barn. All the cows were present in the barn during the samplings.

### 2.2. Instrumentation and Exposure Measurement

From May 2018 to October 2018, 20 weekly measurements (except for only one sample taken in October) of numbers of aerosol particles of different sizes and culturable airborne bacteria were conducted. All the measurements were conducted early in the morning. Samplings were done inside of the barn and in the middle of the central alley between the rows of pens. The numbers of aerosol particles of different sizes were measured by an Optical Particle Sizer (OPS) Model 3330. The various types of airborne bacteria were collected in phosphate-buffered saline (PBS) by a Coriolis *μ* air sampler, and the concentrations of various types of airborne bacteria were measured using the cultural medium sheet. The temperatures and RH were measured with a temperature and RH sensor (RTR-503). Subsequently, statistical analyses of the parameters were conducted to find out the significant differences among various types of airborne bacteria and to determine the relationships among numbers of aerosol particles of different sizes, various types of airborne bacteria, temperatures, and RH.

#### 2.2.1. Measurement of the Aerosol Particle Numbers

The aerosol particle numbers were measured by the Optical Particle Sizer (OPS) Model 3330 (TSI Inc., Shoreview, MN, USA) [29,30,31,32]. The OPS counted the aerosol particle numbers in up to 16 separate channels with a 1-s time resolution and <3000 aerosol particle number concentrations/cm^3^. The aerosol particle numbers were divided into five size fractions: 0.3–0.5 µm, 0.5–1.0 µm, 1.0–2.0 µm, 2.0–5.0 µm, and 5.0–10.0 µm (recommended by International Organization for Standardization) [33]. The OPS was sampled air at 1.0 L/min ± 5% for 20 min (repeat intervals of the OPS set in 2 min where the aerosol particle measurement was 1 min and suspended for 1 min). The total number of aerosol particles was expressed in particles/m^3^.

#### 2.2.2. Collections, Culturing, and Counting of Different Types of Airborne Bacteria

Different types of airborne bacteria were collected using a liquid cyclone air sampler (Coriolis *μ*; Bertin Inc, Montigny-le-Bretonneux, France) [29,30,32,34,35]. A 20-min air sample was collected in 20 mL phosphate-buffered saline with a flow rate of 300 L/min. The samples were then transported to the laboratory using an ice box and cultured immediately.

The concentrations of the various types of airborne bacteria were measured according to the standard plate count method by using the cultural medium sheets (Sanita-kun, JNC Corporation, Tokyo, Japan) as recommended by the JNC Corporation, Japan [29,30,32]. Here, a 1-mL stock sample was diluted to 0.1 M, 0.01 M, and 0.001 M using 10-fold serial dilution. Each diluted sample was inoculated into three culture medium sheets, i.e., total 3 × 3 = 9 culture medium sheets for 1 type of bacteria for different dilutions. After inoculation into the medium sheets, media were incubated 48 h for the airborne total aerobic bacteria and 24 h for the airborne *S. aureus* and *E. coli* at 35 °C. After incubation, characteristic colonies were immediately counted by the colony counter. Among the dilutions, culture medium sheets showing 30–300 colonies were used for calculating the concentrations of various types of airborne bacteria using Equation (1) [32,36]. The principle detection limit of the culture medium sheet method is one colony on each of the two culture medium sheets among three culture medium sheets with 0.1 M diluted sample after incubation. The total number of colonies was expressed as CFU/m^3^.

(1) $$C\;={\;\log}_{10\;}(\frac{N\;\times \;10^{n}}{V_{p}}\;\times \;V_{s\;}\times \;\frac{1}{V_{a}})$$

where *C* is the airborne bacteria concentration (log_10_ CFU/m^3^); *N* is the number of colonies on a culture medium sheet (30–300 colonies); *n* is the serial dilution factor (*n* = 1 for 0.1 M dilution, etc.); $V_{p}$ is the sample volume cultured (1 mL in this study); $V_{s}$ is the total volume of stock sample used for culture (1 mL in this study); and $V_{a}$ is the total volume of air sampled using Coriolis *µ* (6 m^3^ in this study).

#### 2.2.3. Recording of Indoor and Outdoor Temperatures and RH

Over the sampling period, the indoor and the outdoor temperatures and RH were measured with an RTR-503 (T&D Corp., Matsumoto, Japan). The ranges of the temperatures and RH of the RTR-503 were 0–55 °C ± 0.3 °C and 0–95% ± 5%, respectively. Recordings were recorded every hour, and the 24-h mean result was used for analyzing the effects of temperatures and RH on the concentration of the total indoor number of aerosol particles and various types of airborne bacteria.

### 2.3. Statistical Analyses

The statistical analyses were carried out using “R”, a free software environment for statistical computing and graphics (www.r-project.org). For determining the significant differences among different types of airborne bacteria, the means and standard deviations of various types of airborne bacteria were calculated [29,30,32]. The results were considered significant at *p* ≤0.05. The mean concentrations of different sizes of aerosol particles were also calculated, and Spearman correlation tests were utilized for examining the relationships among the various types of airborne bacteria, aerosol particle numbers, temperatures, and RH, with *p* ≤ 0.05 and R values ≥0.40 being significant [30,32]. The higher R values (almost 1.00) and lower *p* ≤ 0.05 were considered as strong associations and vice versa.

## 3. Results

### 3.1. The Concentrations of Aerosol Particles of Different Sizes during the Summer Season

**Figure 2:**
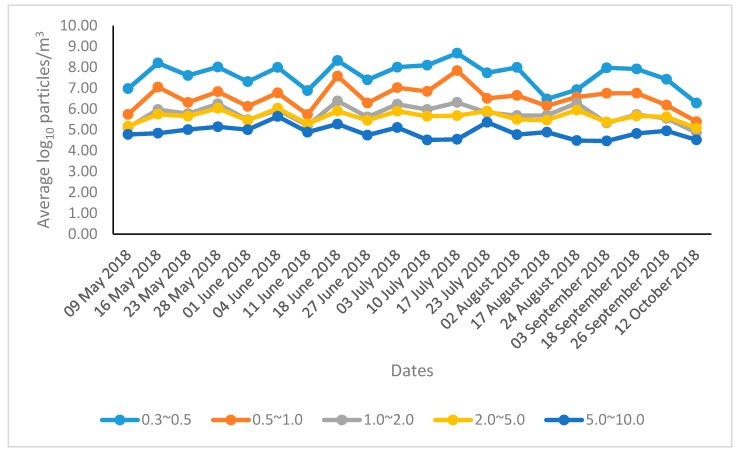
The daily average concentrations of aerosol particles of different sizes.

The mean concentrations of aerosol particle sizes 0.3–0.5, 0.5–1.0, 1.0–2.0, 2.0–5.0, and 5.0–10.0 µm during the summer season were 7.61, 6.56, 5.76, 5.63 and 4.89 log_10_ particles/m^3^, respectively. The highest concentrations of 0.3–0.5, 0.5–1.0, 1.0–2.0, and 2.0–5.0 µm aerosol particles during the summer season were 8.13, 7.05, 6.09, and 5.78 log_10_ particles/m^3^, respectively, observed in July, and the highest concentration for 5.0–10.0 µm aerosol particles was 5.11 log_10_ particles/m^3^, observed in June. The daily average concentrations of aerosol particles of different sizes are shown in Figure 2.

### 3.2. The Concentrations of Different Types of Airborne Bacteria during the Summer Season

**Figure 3:**
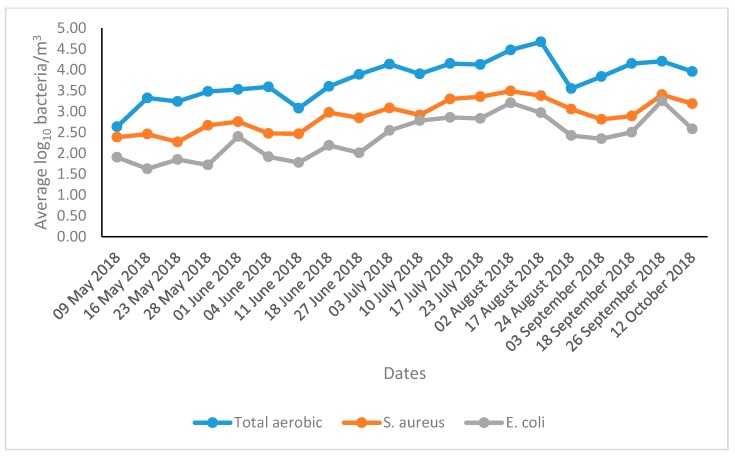
The daily average concentrations of different types of airborne bacteria. *S. aureus: Staphylococcus aureus*; *E. coli: Escherichia coli*.

The mean concentrations of airborne total aerobic bacteria, *S. aureus*, and *E. coli* were 3.78, 2.91, and 2.39 log_10_ CFU/m^3^, respectively. The highest concentrations of airborne total aerobic bacteria, *S. aureus*, and *E. coli* were 4.23, 3.31, and 2.87 log_10_ CFU/m^3^, respectively, observed in August. The daily average concentrations of different types of airborne bacteria are shown in Figure 3.

**Figure 4:**
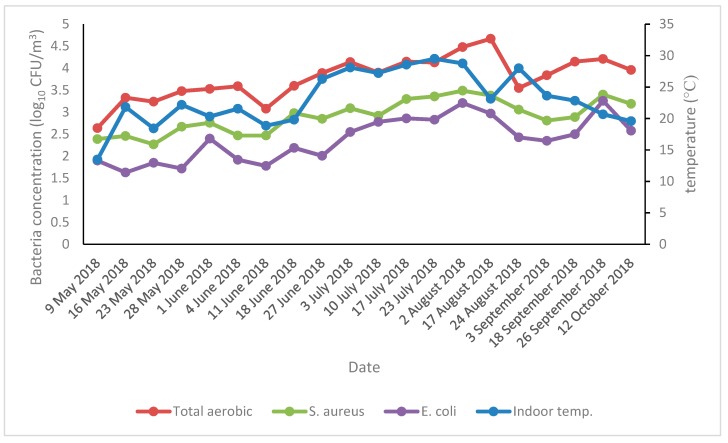
The concentrations of various types of airborne bacteria and air temperatures.

The concentrations of airborne total aerobic bacteria, *S. aureus*, and *E. coli* were dependent on air temperatures, i.e., the high air temperatures were related to the high concentrations of airborne total aerobic bacteria, *S. aureus*, and *E. coli*. However, these concentrations fluctuated with fluctuations in air temperatures, and the concentrations gradually decreased with decreasing air temperatures (Figure 4).

**Figure 5:**
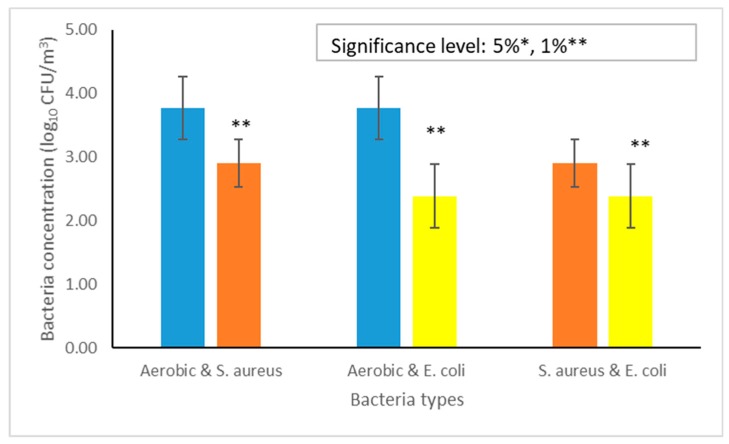
The average concentrations of different types of airborne bacteria during the summer season.

The airborne total aerobic bacteria concentrations were significantly different (*p* < 0.01) compared with the airborne *S. aureus* and *E. coli*. The airborne *S. aureus* concentrations were significantly different (*p* < 0.01) compared with the airborne *E. coli* (Figure 5).

### 3.3. The Environmental Factors Inside and Outside of the Dairy Barn during the Summer Season

The mean temperature and RH during the summer season inside of the barn were 23.14 °C and 75.36%, respectively. The mean temperature and RH during the summer season outside of the barn were 22.05 °C and 79.01%, respectively. Daily mean temperatures and RH (inside and outside of the barn) during the summer season are shown in Table 1.

### 3.4. The Interrelationships among the Aerosol Particle Numbers, Various Types of Airborne Bacteria, Temperatures, and RH

#### 3.4.1. Relationships between Numbers of Aerosol Particles of Different Sizes and Different Types of Airborne Bacteria

In the present study, relationships between numbers of aerosol particles of different sizes and different types of airborne bacteria were not found.

#### 3.4.2. Relationships between Environmental Factors and Numbers of Aerosol Particles of Different Sizes

The relationships between environmental factors (outdoor and indoor) and numbers of aerosol particles of different sizes are shown in Table 2.

#### 3.4.3. Relationships between Environmental Factors and Different Types of Airborne Bacteria

**Figure 6:**
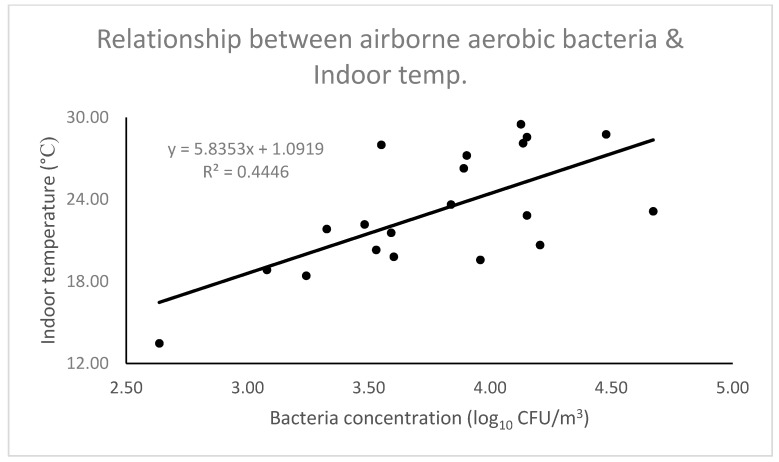
Relationship between the airborne total aerobic bacteria and indoor temperatures.

The relationships between environmental factors (outdoor and indoor) and different types of airborne bacteria are shown in Table 3. A total of six relationships with different types of airborne bacteria and outdoor and indoor temperatures were found. The relationship between indoor temperatures and airborne total aerobic bacteria is shown in Figure 6. A relationship between RH (outdoor and indoor) and different types of airborne bacteria was not found.

## 4. Discussion

In the present study, the aerosol particle numbers were divided into five categories based on sizes. The knowledge of size distributions of the aerosol particles in livestock barns is conducive to understanding transport behaviors of the aerosol particles and health risk to the animals and humans, and to improving the quality of the indoor air.

The present findings indicate that aerosol particle number concentrations for all sizes remain relatively high during the whole summer season, with the fine aerosol particle number (0.3–2.0 µm) concentrations being higher (Figure 2). Previously, the mean concentrations of aerosol particle sizes 0.3–0.5, 0.5–1.0, 1.0–2.0, 2.0–5.0, and 5.0–10.0 µm detected in a swine barn during the summer season were 7.93, 6.83, 5.93, 5.84, and 5.44 log_10_ particles/m^3^, respectively [30]. Mean concentrations of 7.60, 6.59, 5.77, 5.69, and 4.99 log_10_ aerosol particles/m^3^, respectively, were detected in a dairy calf barn [32], and mean concentrations of 7.84, 6.75, 5.85, 5.71, and 5.11 log_10_ aerosol particles/m^3^, respectively, were detected in an open-type dairy barn [29], consistent with our present findings. These higher concentrations of the fine aerosol particles can penetrate deeply into the body tissues of the animals and workers in the livestock farms. They may cause respiratory diseases, cardiovascular diseases, type 2 diabetes, and even cancers [37,38]. Therefore, mitigation of these aerosol particles should be a high priority for the improvement of the quality of the indoor air.

The present findings indicate that the concentrations of all types of airborne bacteria were very high in the tie-stall dairy barn during the summer season (Figure 3). The present findings also showed that concentrations of all types of airborne bacteria started to increase from May and remained higher up to August. From September, the concentrations of all types of airborne bacteria began to decrease and are in line with temperature fluctuations (Figure 4). Previously the mean concentrations of airborne total aerobic bacteria, *S. aureus*, and *E. coli* were 4.15, 3.33, and 3.02 log_10_ CFU/m^3^, respectively, detected during the summer season in a dairy calf barn [32]. Concentrations of 3.93, 3.51, and 2.01 log_10_ CFU/m^3^, respectively, were detected during the summer season in a swine barn [30], consistent with our present findings. The ambient air temperatures had a stimulating impact on the concentration level of airborne bacterial concentrations. On the other hand, higher temperatures contribute to the higher levels of airborne microorganisms, providing viability for their growth [39,40], which is consistent with our present findings.

The total aerobic bacteria at the present study consists of many pathogenic bacteria (Figure 5). In the present study, as a single species the concentration of the airborne *S. aureus* was relatively high. The most probable reason for these higher concentrations of the airborne *S. aureus* in the livestock environment is due to their multiple routes of shedding from the animal species such as bedding materials, skin, respiration, floor materials, and fecal excretion. The occurrence of relatively lower concentrations of the airborne *E. coli* in the dairy barns is because of their principal route of shedding through fecal excretion [19].

In the present study, relationships between the numbers of aerosol particles of different sizes and various types of airborne bacteria were not observed. Generally, microorganisms bind with the aerosol particles and spread to the surrounding environment. Previous studies found relationships between the aerosol particles and the airborne bacteria [30,41,42,43]. A probable reason for the different findings in the present study may be due to the different microenvironments studied.

Airborne bacteria usually are associated with the aerosol particles in the livestock housing environments [44], and aerosol particles act as a carrier of a large variety of microorganisms [4,45,46]. The aerosol particles larger than 2.0 μm in diameter were found to carry high amounts of bacteria in the livestock barns [45,47]. It is speculated that the aerosol particles with larger aerodynamic size and higher mass contain more bacteria [44]. The more aerosol particles are suspended in the air, the more bacteria exist in the air [44]. The linear relationships between the airborne bacteria concentrations and the aerosol particles’ mass concentrations varied and could be affected by several factors such as the air temperatures, RH, and sources of aerosol particles.

In the present study, more significant positive relationships (three relationships) between aerosol particle numbers and outside temperatures (high temperatures relates to high concentrations of the aerosol particle numbers) were found compared to inside temperatures (two relationships) (Table 2). In higher temperatures, there is a higher suspension of dispersed and drier aerosol particle numbers from the feed, litter, manure, soil, and floor materials in the air of dairy barns, as evidenced by a previous study [28]. It was also evidenced that temperatures have a significant, positive association with biological aerosol concentrations [48], which supports our present findings. Conversely, relationships between RH (outside and inside) and numbers of aerosol particles of different sizes were not found, which indicates that the RH has no regulating effects on the aerosol particle numbers in the tie-stall dairy barn during the summer season. A previous study has reported the relationship between airborne bacteria and RH [49] in the laying hen house, which is different from our present findings. The most likely reason for these different findings may be due to the differences in the species of animals. The previous study also showed that RH influences water evaporation from airborne particles, and thus their density and diameter. RH also affected their settling velocity [21], which is inconsistent with our present study.

In the present study, relatively stronger positive relationships (higher R-value and lower *p*-value) between the various kinds of airborne bacteria and the indoor temperatures (high temperatures relates to a high concentrations of the various types of airborne bacteria) were found rather than the outdoor temperatures and the different types of airborne bacteria (Table 3). These were may be due to the higher temperatures inside the dairy barn than outside of the dairy barn (the temperatures inside the dairy barn were 1.09 °C greater than the outside temperatures). Previously, relationships between the airborne bacteria and temperatures were also reported in other studies [27,49], which supports our present findings. The level of concentrations of microorganisms in the air is mostly determined by parameters such as temperature and RH, which determine the appropriate conditions for microorganisms to grow colonies [50]. The present study also showed that air temperatures have profound effects on the various types of airborne bacteria.

A previous study showed that the atmospheric temperatures had a significant positive influence on microbial activity, whereas RH had no significant influence [51], which is in agreement with our present study. The concentrations of the microorganisms in the air varies at different times and sites [52,53,54,55]. According to prior research, temperatures always affect microbial concentrations, but the effects of the RH vary with the geographic region [51], which supports our present findings.

## 5. Conclusions

The present study investigates aerosol number concentrations, concentrations of the various airborne bacteria, temperatures, and RH in a tie-stall dairy barn during the summer season to evaluate the relationships among them. Relatively higher concentrations of all aerosol particles of different sizes and different types of airborne bacteria were found in the tie-stall dairy barn during the summer season. Relationships between numbers of aerosol particles of different sizes and various types of airborne bacteria were not found during the summer season. The profound effects of temperatures on the numbers of aerosol particles of different sizes and the different types of airborne bacteria were found. Relationships of RH with sizes of aerosol particles or various types of airborne bacteria were not found.

One of the main limitations of the present study is that the airflow within the barn was not measured. Future studies should be undertaken for finding the effects of airflow on numbers of aerosol particles of different sizes and various types of airborne bacteria. For finding the full seasonal variation of the numbers of aerosol particles of different sizes and different types of airborne bacteria, a year-round study should also be conducted. As the present study analyzes samples from the same time of the day, future studies should also undertake for finding the diurnal variations of the different sizes of the aerosol particle numbers and various types of airborne bacteria with samplings from the different times of the day.

The present findings will be crucial to the design of experiments aimed at measuring aerosol particles and various types of pathogenic airborne bacteria from the perspective of public health in the dairy barns. The present results will also help to explore ways to control the aerosol particles and various types of airborne bacteria in the indoor air in dairy barns and ultimately will help to reduce environmental pollution and transmission of various infectious diseases from dairy barns.

## Figures and Tables

**Table 1:** The indoor and outdoor temperatures and relative humidity (RH) of the dairy barn over the sampling period. Temp: temperature.

Date	Indoor		Outdoor	
	Temp.	RH	Temp.	RH
9 May 2018	13.48	74.78	11.68	81.06
16 May 2018	21.83	69.77	21.27	71.08
23 May 2018	18.42	74.94	17.75	74.94
28 May 2018	22.18	76.26	21.32	79.09
1 June 2018	20.31	64.88	19.65	66.78
4 June 2018	21.56	66.52	20.92	68.22
11 June 2018	18.85	85.01	17.69	91.03
18 June 2018	19.81	82.94	18.40	89.11
27 June 2018	26.28	78.31	25.51	80.92
3 July 2018	28.11	72.90	27.44	74.56
10 July 2018	27.22	81.36	26.08	86.09
17 July 2018	28.56	81.38	27.74	84.29
23 July 2018	29.50	66.91	28.40	76.85
2 August 2018	28.76	73.72	28.04	75.58
17 August 2018	23.13	54.19	22.54	53.74
24 August 2018	28.00	81.51	27.06	84.15
3 September 2018	23.63	87.96	22.07	94.08
18 September 2018	22.83	84.84	21.24	90.19
26 September 2018	20.67	74.92	18.48	79.86
12 October 2018	19.58	74.13	17.69	78.64
Mean ± SD	23.14 ± 4.31	75.36 ± 8.17	22.05 ± 4.53	79.01 ± 9.55

**Table 2:** Relationships between environmental factors and numbers of aerosol particles of different sizes.

Aerosol Size	Indoor Temp.	Outdoor Temp.	Indoor RH	Outdoor RH
0.3−0.5 µm	-	-	-	-
0.5−1.0 µm	R = 0.49, *p* = 0.03	R = 0.51, *p* = 0.02	-	-
1.0−2.0 µm	R = 0.52, *p* = 0.02	R = 0.55, *p* = 0.01	-	-
2.0−5.0 µm	-	R = 0.45, *p* = 0.04	-	-
5.0−10.0 µm	-	-	-	-

**Table 3:** Relationships between environmental factors and different types of airborne bacteria.

Bacteria Type	Indoor Temp.	Outdoor Temp.	Indoor RH	Outdoor RH
Total aerobic	R = 0.67, *p* = 0.00	R = 0.64, *p* = 0.00	-	-
*S. aureus*	R = 0.62, *p* = 0.00	R = 0.58, *p* = 0.00	-	-
*E. coli*	R = 0.52, *p* = 0.02	R = 0.47, *p* = 0.04	-	-
